# Malingering Extraordinary

**Published:** 1872-04

**Authors:** 


*Malingering Extraordinary.*

An instance of feigned disease which, as regards skill in simulation and successful execution, may be considered unique, has recently transpired in London, and is reported at length in the *Lancet* for February 17th.

The subject was a well-educated and intelligent man of 43. He usually assumed the *role* of a physician, and generally gave a straightforward history of his case for the time being and of his social antecedents. On one occasion his reputed relationship to Dr. Marshall Hall passed unchallenged. He successfully duped eleven of the hospital surgeons and physicians in London, some of them men of eminence. His object in carrying out the deception was never discovered. He passed successively from one hospital to another, remaining sometimes till he was pronounced convalescent, at other times taking sudden leave when suspicion appeared to be aroused to a degree too unpleasant.

His success at simulating was confined to the graver nervous affections, a department of pathology one would think ill adapted to malingering. He was treated for tetanus, for hemiplegia, and for ingravescent apoplexy, and his imitations of symptoms were so perfect as to entirely blind his medical attendants. He learned at one hospital points in his disease to be improved upon at the next; his tongue was at one time protruded too straight to conform to the paralytic condition he otherwise presented so well; at the next stopping place the lingual deviation was correctly assumed. At one time he suffered from traumatic tetanus, but the surgeons could find no cicatrix about the scalp to recall the alleged fall of forty feet some years before; the next time he has tetanus there is a distinct scar. His temperature arose once to 102° Fah., as it should; it was some time subsequently discovered that he had slyly placed the thermometer bulb near the candle flame when it should have been in his axilla. His first attempts at opisthotonos were wanting in rigidity of the abdominal muscles; he profited by the suggestion, and at the next hospital his tetanus was attended with spasms which made his abdominal muscles "as hard as boards." Night or day, he never forgot to carry out the simulated symptoms. In one hospital he had tetanus for ten days, and although a carbuncle, *which he did not feign*, came on the back of the neck, and was freely opened without an anæsthetic, the tetanic opisthotonos was not meantime neglected.

Treatment did not discourage him, and the variety of therapeutics to which he was subjected was heroic, and was heroically endured. Opium and morphia were administered by the stomach and the rectum and under the skin. Calabar bean, belladonna, bromide, and iodide of potassium, chloroform and hydrate of chloral, were given in enormous doses to control his paroxysms. Ice-bags and ether-spray to the spine were also duly tried. He was watched with at night by diligent students enthusiastic to study the natural progress of tetanus; he was made the subject of a clinical lecture on "arachnoid hæmorrhage" before a medical class; and the notes of his case in the hospital case-books were always voluminous as the urgency of his disease appeared to demand.

One one occasion, he was believed to be incurable; a solicitor was sent for, and the pseudo-doctor made his will bequeathing a handsome sum to the assistant physician and to the hospital; this thoughtfulness on his part resulted in special comforts from the hospital authorities, including the best of wines and of food.

The period of this arch impostor's performances extended over nearly four years, and under his successive aliases comprised such hospitals as St. Bartholomew's, Middlesex, and St. George's.

The *Lancet* remarks editorially on this case: "How many hospital statistical tables he must have falsified in his time! What a God-send such a patient would prove to the man with a firm faith in some new theory of disease and bran-new remedy for its cure!" —*Boston Med. and Surg. Journal.*

